# Editorial: Rising stars in neurosurgery 2025

**DOI:** 10.3389/fsurg.2025.1641348

**Published:** 2025-07-09

**Authors:** Kevin K. Kumar, Redi Rahmani, Ignatius Esene, Hemmings Wu, Martin N. Stienen

**Affiliations:** ^1^ Department of Neurosurgery, Dell Medical School, The University of Texas at Austin, Austin, TX, United States; ^2^ Department of Neurosurgery, University of Louisville, Louisville, KY, United States; ^3^ Neurosurgery Division, Faculty of Health Sciences, University of Bamenda, Bambili, Cameroon; ^4^ Department of Neurosurgery, Zhejiang University School of Medicine Second Affiliated Hospital, Hangzhou, China; ^5^ Department of Neurosurgery & Interdisciplinary Spine Center, H-OCH Health Ostschweiz, Kantonsspital St.Gallen & University of St.Gallen, St.Gallen, Switzerland

**Keywords:** neurosurgery, training, mentorship, innovation, brain, spine

**Editorial on the Research Topic**
Rising stars in neurosurgery 2025

A primary mission of academic neurosurgery is to identify, support, and amplify the voices of emerging investigators in a specialty defined by technical mastery, intellectual rigor, and a uniquely long and demanding training pathway. This special issue of *Frontiers in Surgery - Rising Stars in Neurosurgery 2025*, seeks to highlight young neurosurgeons who pursue research alongside their surgical practice. This issue's contributors include residents, fellows, and early-career faculty from institutions across the globe. Their work reflects the breadth and depth of contemporary neurosurgical inquiry, spanning pediatric epilepsy, vascular anomalies, neuroinflammation, spinal biomechanics, and skull base neurosurgery. Together this work demonstrates neurosurgery's increasing reliance on translational science, precision diagnostics, and data-driven decision making. Moreover, the new age of artificial intelligence has arrived, and with it comes a profound shift in how neurosurgeons diagnose, prognosticate, and treat disease. From predictive analytics in perioperative care to molecular profiling of tumors and vascular malformations, the next generation of neurosurgeons will increasingly rely on artificial intelligence to personalize treatment and enhance clinical decision-making.

For example, two contributions in pediatric neurosurgery exhibit this shift. The first represents institutional experience with intracranial neuromodulation in children with drug-resistant epilepsy by Uchitel et al. While initial trials utilizing deep brain stimulation and responsive neurostimulation excluded pediatric patients, important insights can be gleaned to achieve seizure control in this vulnerable population. This represents a key step both for safety and optimizing neurodevelopmental trajectory. A companion study by Fariyike et al. focuses on diagnostic challenges in pediatric epilepsy with focus on the role of somatic mutation in small cortical regions (Fariyike et al.). Using advanced molecular techniques, the authors reframe epilepsy as a genetic mosaic disorder. This perspective allows neurosurgeons to work as diagnostic collaborators, prior to resection or ablation, defining the underlying biology of the disease.

In cerebrovascular neurosurgery, an important article by Jabarkheel et al. explores the molecular underpinnings of brain arteriovenous malformations (AVMs) (Jabarkheel et al.). They present compelling evidence of the role of the KRAS/MAPK signaling pathways. This concept expands the definition of AVMs from purely anatomical perspective, allowing for early pharmacological intervention that could displace current treatment paradigms of open surgery, endovascular embolization, or radiosurgery. This innovation at the intersection of molecular biology and vascular neurosurgery shows promise.

Outside of the basic science laboratory, continual improvement of neurosurgical operative techniques is essential. A study by Padmanaban et al. regarding carotid endarterectomy performed under regional anesthesia shows how thoughtful improvement of classic approaches can improve patient safety and outcomes (Padmanaban et al.). In an aging population with an increasing number of comorbidities, regional techniques allow for precise neurologic monitoring and less hemodynamic stress. Similarly, Fischer et al. compare lateral lumbar interbody fusion (LLIF) with release of the anterior longitudinal ligament (so-called “anterior column realignment”) vs. standard LLIF techniques using expandable spacers (Fischer et al.). Their retrospective cohorts study provides valuable guidance for patients requiring complex deformity correction. As spine instrumentation becomes increasingly modular and patient-specific, this work helps refine surgical decision-making and support the personalization of care.

Another innovative article by Levinson et al. highlights advances in our understanding of the role of neuroinflammation in acute ischemic stroke (Levinson et al.). The authors synthesize recent evidence on microglial activation, cytokine cascades, and peripheral immune interactions. Their discussion offers potential targets for therapeutic intervention and calls for a broader understanding of stroke as a neuroimmunologic event rather than a purely vascular problem.

The issue also includes a study on risk prediction following endoscopic transsphenoidal resection of pituitary adenomas by Wang et al. Here the authors seek to exemplify the role of predictive analytics in perioperative management in skull base surgery. Modeling surgical risk preoperatively will likely become an integral part of the neurosurgical workflow as machine learning techniques allows greater leveraging of electronic health records and preoperative data.

Beyond the scientific content, the most striking feature of this collection may be the academic mentorship exhibited among the contributors. The senior authors serve as mentors, collaborators, and sponsors for the rising stars featured here. Their role in fostering early-career development is critical. In a field where time is scarce and clinical productivity is often prioritized; meaningful mentorship is a strategic investment in the future of the discipline. These partnerships are not incidental; they are foundational to the survival of the neurosurgeon-scientist model. The international representation in this issue is equally noteworthy. Contributors hail from institutions in the United States, Europe, Africa and Asia, reflecting a growing recognition that academic neurosurgery must be inclusive and globally engaged.

The long-term viability of academic neurosurgery requires institutional structures that support inquiry: protected research time, funding mechanisms for early-career investigators, and recognition of academic contributions in promotion pathways. Initiatives such as *Frontiers in Surgery* - *Rising Stars in Neurosurgery* are essential in creating visibility and momentum, but they must be matched by sustained commitment from departments, institutions, and funding agencies.

This year's issue reflects what is possible when early-career neurosurgeons are supported, challenged, and empowered to lead. The work presented here advances a model of neurosurgery that is not only technically excellent, but innovative and pushing the frontiers of translational science.

